# How Long Are Reperfusion Therapies Beneficial for Patients after Stroke Onset? Lessons from Lethal Ischemia Following Early Reperfusion in a Mouse Model of Stroke

**DOI:** 10.3390/ijms21176360

**Published:** 2020-09-02

**Authors:** Takayuki Nakagomi, Yasue Tanaka, Nami Nakagomi, Tomohiro Matsuyama, Shinichi Yoshimura

**Affiliations:** 1 Institute for Advanced Medical Sciences, Hyogo College of Medicine, 1-1 Mukogawacho, Nishinomiya 663-8501, Japan; s-yoshi@hyo-med.ac.jp; 2 Department of Therapeutic Progress in Brain Diseases, Hyogo College of Medicine, 1-1 Mukogawacho, Nishinomiya 663-8501, Japan; tomohiro@hyo-med.ac.jp; 3 Department of Neurosurgery, Hyogo College of Medicine, 1-1 Mukogawacho, Nishinomiya 663-8501, Japan; yasuet@hyo-med.ac.jp; 4 Department of Surgical Pathology, Hyogo College of Medicine, 1-1 Mukogawacho, Nishinomiya 663-8501, Japan; na-nakagomi@hyo-med.ac.jp

**Keywords:** ischemic stroke, reperfusion, histopathology, neural cells, vascular cells

## Abstract

Ischemic stroke caused by cerebral artery occlusion induces neurological deficits because of cell damage or death in the central nervous system. Given the recent therapeutic advances in reperfusion therapies, some patients can now recover from an ischemic stroke with no sequelae. Currently, reperfusion therapies focus on rescuing neural lineage cells that survive in spite of decreases in cerebral blood flow. However, vascular lineage cells are known to be more resistant to ischemia/hypoxia than neural lineage cells. This indicates that ischemic areas of the brain experience neural cell death but without vascular cell death. Emerging evidence suggests that if a vascular cell-mediated healing system is present within ischemic areas following reperfusion, the therapeutic time window can be extended for patients with stroke. In this review, we present our comments on this subject based upon recent findings from lethal ischemia following reperfusion in a mouse model of stroke.

## 1. Introduction

Ischemic stroke is a critical disease caused by the occlusion of cerebral arteries. Fortunately for patients, reperfusion therapies such as recombinant tissue plasminogen activator (t-PA) and neuroendovascular treatments [1,2,3], have led to tremendous advances in clinical outcomes. Presently, the application of reperfusion therapies is determined by neuroimaging like computed tomography and magnetic resonance imaging. In animal brains, real-time in vivo imaging by two-photon microscopy enables image acquisition of dynamic cell-cell interactions [4,5]. In human brains, although neuroimaging evaluating ischemic lesions and/or the neurovascular unit (NVU) has advanced considerably [6,7,8,9,10,11], it cannot fully assess metabolic processes within ischemic areas [12]. Thus, the utility of reperfusion therapies for the treatment of patients with stroke warrants discussion beyond the scope of neuroimaging.

Upon receiving reperfusion therapies, patients with stroke will often recover through the rescue of mature neural cells that have survived, despite decreases in blood flow. However, because of sensitivities to ischemic insults, mature neural cells such as neurons and glia, will rapidly undergo cell death if they are not rescued immediately following stroke onset [13,14,15]. In contrast, vascular lineages, such as endothelial cells and pericytes, are very resistant to ischemia and can survive for several days even within ischemic areas [13,16].

Under normal (non-ischemic) conditions, vascular lineages (endothelial cells and pericytes) together with neural lineages (neurons and glia) constitute the NVU and play an important role in maintaining this unit [17,18]. Although the precise roles of vascular cells that survive within ischemic areas under pathological conditions remains unclear, increasing evidence shows that these cells have the potential to promote brain repair by functioning as nursing cells [19,20], stem cells [21,22], and stem cell niches [23,24]. Thus, although reperfusion therapies after stroke have focused on rescuing neural cells, vascular lineage cells may represent novel targets of these treatment options. In this review, we introduce our comments based on recent studies performed after early reperfusion under lethal ischemia in a mouse model of stroke [16,25].

## 2. Histopathological Stages Following Ischemic Stroke

**Figure 1:**
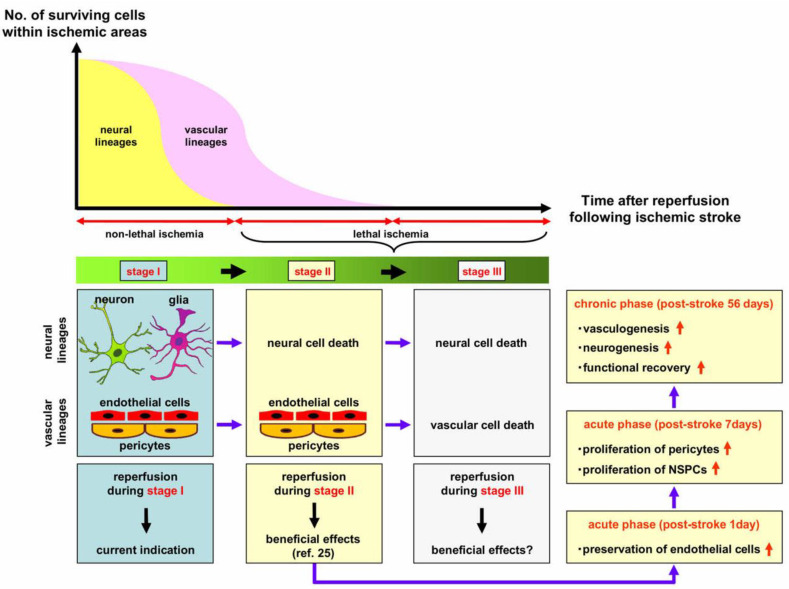
Proposed histopathological stages within ischemic areas. After onset of ischemic stroke, the histopathology within ischemic areas can be categorized into three stages as the time from stroke onset advances: non-lethal ischemia without neural or vascular cell death despite the presence of decreased blood flow (stage I), lethal ischemia with neural cell death but without vascular cell death (stage II), and lethal ischemia with neural and vascular cell death (stage III). Reperfusion in stage II has the potential to provide advantageous effects via the mechanism suggested in the main text of this review. NSPCs—neural stem/progenitor cells.

Histologically, brains consist of the NVU, which is composed of neural (neurons and glia) and vascular (endothelial cells and pericytes) lineage cells [26]. Neural lineages, including neurons and glia, are sensitive to the ischemia/hypoxia occurring after stroke; however, there is increasing evidence that vascular lineages, including endothelial cells and pericytes, are more resistant to such insults [13,14,16]. Therefore, histopathologically, brain tissues within ischemic areas can be split into at least three stages as the time after stroke onset advances: non-lethal ischemia with no neural and vascular cell death despite decreased cerebral blood flow (CBF) (stage I), lethal ischemia with neural cell death but without vascular cell death (stage II), and lethal ischemia with neural and vascular cell death (stage III) (Figure 1).

As an intervention therapy following ischemic stroke, prior studies have focused on the rescue of cells (in particular, mature neurons) that survive despite decreased blood flow (stage I in Figure 1). Nevertheless, as mentioned earlier, vascular lineages are more resistant to ischemia than neural lineages. In addition, not only neural lineages, including neurons [27] and glia [28], but also vascular lineages [23] are correlated with brain function. Thus, even after lethal ischemia with mature neural cell death (stage II in Figure 1), vascular lineages within ischemic areas can still function as a cell niche to promote healing processes, thereby having the potential to contribute to functional recovery. Although the application of reperfusion therapies has expanded, only a small population of patients with stroke are eligible for such therapies due to factors such as the limited therapeutic time window, which benefits patients in stage I. However, if reperfusion during stage II is advantageous for patients, more can receive its benefits because of the enlarged therapeutic time window.

## 3. Findings after Early Reperfusion under Lethal Ischemia in a Mouse Model of Stroke

### 3.1. Reperfusion and Stage II

Recently, Tachibana et al. demonstrated that reperfusion following lethal ischemia had beneficial effects such as neurological functional recovery in a mouse model of stroke [16]. More recently, with the use of a highly reproducible mouse model of middle cerebral artery occlusion (MCAO) [14,29,30], we investigated whether reperfusion during stage II can have beneficial effects at acute (1–7 days after MCAO) and chronic (more than 3 weeks after MCAO) periods by focusing on histology and brain function [25].

**Figure 2:**
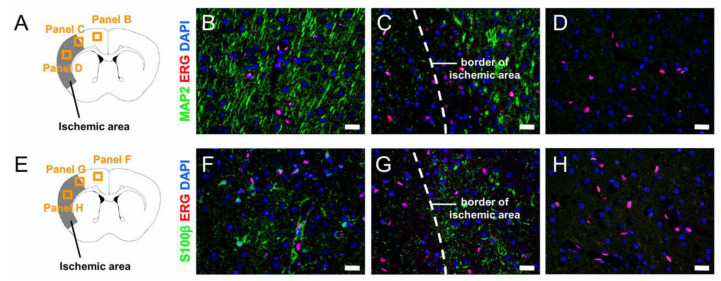
Histopathological findings of mouse brains in stage II. Although immunohistochemistry at post-stroke day 1 showed that reperfusion after ischemia with a duration of 90 min induced cell death of neurons (MAP2^+^ cells) (**A**–**D**) and glia (S100β^+^ astrocytes) (**E**–**H**) within ischemic areas, some endothelial cells (ERG^+^ cells) (**A**–**H**) survived (MAP2 (**B**–**D**: green), ERG (**B**–**D**: red), DAPI (**B**–**D**: blue); S100β (**F**–**H**: green), ERG (**F**–**H**: red), DAPI (**F**–**H**: blue)). Scale bars = 20 µm (**B**–**D**,**F**–**H**). ERG—ETS-related gene; MAP2—microtubule-associated protein 2.

Histopathologically, H&E staining at the acute period (post-stroke day 1) showed that reperfusion after ischemia with a duration of 90 min can induce cell death within ischemic areas. Moreover, immunohistochemical analysis at post-stroke day 1 demonstrated that reperfusion after ischemia with a duration of 90 min did not rescue mature neural cells, such as neurons and glia (Figure 2). These findings indicate that stage I had already passed, even if reperfusion was performed after ischemia with a duration of 90 min. However, some endothelial cells were preserved within ischemic areas (Figure 2), suggesting that reperfusion was being performed during stage II.

### 3.2. Reperfusion and Endothelial Cells

In normal brains, endothelial cells constitute the blood–brain barrier (BBB), which is formed by interendothelial junctions, including tight and adherens junctions. Endothelial cell-to-cell junctions regulate and maintain functions of intercellular adherens, including cell growth and blood vessel formation [31]. However, in brains under pathological conditions such as after ischemic stroke, endothelial cell-to-cell junctions are loosened and BBB dysfunction induces increased permeability of blood vessels [32,33]. Previous studies using rodent stroke models showed that BBB leakage peaks during acute periods (1–7 days after ischemic stroke) [34].

Consistent with these reports, our recent study showed that BBB leakage assessed by albumin expression level was significantly greater in ischemic areas than in contralateral non-ischemic areas. These findings were not different between reperfusion after ischemia with a duration of 90 min and permanent ischemia at post-stroke days 1 and 3. However, compared with permanent ischemia, reperfusion after ischemia with a duration of 90 min reduced BBB permeability at post-stroke day 7 [25]. Consistent with this observation, a previous study showed that early reperfusion improved BBB permeability, as assessed by T2* perfusion-weighted source images, in patients with stroke during acute periods [35]. Although the mechanism remains unclear, we found that, in comparison with permanent ischemia, reperfusion after ischemia with a duration of 90 min significantly preserved endothelial cells within ischemic areas at post-stroke days 1 and 7 [25]. In addition, mild BBB disruption in patients with stroke was reversible by early reperfusion [35]. Thus, it is possible that reperfusion following ischemia may simply prevent endothelial cells from falling into cell death.

Of interest, consistent with a previous study [16], we found that larger numbers of neutrophils were present within ischemic areas after permanent ischemia compared with those of reperfusion after ischemia [25]. It has been documented that anoxia/reperfusion injuries promote the adherence of neutrophils to endothelial cells in vitro and that several cell adhesion molecules (e.g., intercellular adhesion molecule 1, vascular cell adhesion molecule 1, E-selectin, and P-selectin) play an important role in neutrophil migration into endothelial cells [31,36,37]. Therefore, compared with reperfusion following ischemia, these molecules may be more activated under permanent ischemia. Activated neutrophils following ischemic stroke induce BBB breakdown, in part via producing matrix metalloproteinases (MMPs) which cause injury of blood vessels [31,34,38]. In addition, neutrophil elastase was reported to increase the permeability of blood vessels [39]. Therefore, although the precise mechanism by which neutrophils regulate BBB permeability is unclear [40], it is possible that, compared with reperfusion after ischemia, permanent ischemia promotes BBB disruption via endothelial injuries by neutrophils.

BBB dysfunction induces not only increased permeability of blood vessels, but also hemorrhagic transformation in the brain [34]. Previous studies showed that BBB loosening by reperfusion therapies such as t-PA treatment was related to the occurrence of hemorrhagic transformation [35,41,42,43,44]. However, our recent study demonstrated that early reperfusion did not increase the incidence of hemorrhagic transformation during the periods observed up to 8 weeks after MCAO [25]. Although we do not know the reason for this discrepancy, other recent studies also revealed concrete evidence that endothelial cells were more maintained in mice after early reperfusion compared with mice without reperfusion (permanent ischemia) [16,25]. Thus, several factors (e.g., time until reperfusion after ischemic stroke, reperfusion area, and age) may account for these differences between findings.

It remains unclear whether damaged blood vessels can reconstruct the BBB following ischemic stroke. Previous studies found that the traits of endothelial cells were altered following injury (i.e., transdifferentiating into fibroblastic-like cells via endothelial-mesenchymal transition (endMT)) [45]. Although a recent study showed that reperfusion after ischemia causes endMT in brain [46], our previous study using genetic mapping for the endothelial cell marker VE-cadherin showed that endothelial cells within and around ischemic areas did not transform into fibroblastic-like cells, and that they maintained expression of the endothelial marker during the acute phase [13]. In that study, we also found that expression of VE-cadherin was increased at both the promoter and protein level within ischemic areas. These findings indicate that endothelial cells, which survived at the site of ischemic areas, can proliferate following ischemic stroke. In further support of this conclusion, we showed that the number of endothelial cells at peri-ischemic areas increased during chronic periods (post-stroke day 56) compared with those observed during acute periods (post-stroke days 1 and 7) [25]. Although these findings were observed both in reperfusion after ischemia with a duration of 90 min and permanent ischemia, the number of endothelial cells were significantly greater in reperfusion after ischemia. Therefore, though the precise roles of endothelial cells following ischemic stroke remain unclear, increased endothelial cells following early reperfusion may contribute to reconstruction of the BBB, thereby decreasing BBB permeability.

### 3.3. Reperfusion and Pericytes

Together with endothelial cells, pericytes, a key component of the NVU, also have an important part in BBB maintenance by regulating endothelial and astrocytic function [17]. In mice, pericyte ablation promotes BBB permeability and causes BBB disruption [17,47]. During angiogenesis, pericytes and endothelial cells cooperatively play an essential role in forming and stabilizing new blood vessels via several signaling factors, such as platelet-derived growth factor subunit B (PDGFB)/platelet-derived growth factor receptor-beta (PDGFRβ), transforming growth factor β, Notch, and vascular endothelial growth factor [48].

Compared with permanent ischemia, reperfusion during stage II decreases the size of the ischemic area at post-stroke day 7 [25]. Although the precise mechanism underlying these effects is not fully understood, reperfusion during this stage dramatically promotes proliferation of brain pericytes, which are known to have an important role in wound healing following injuries like ischemic stroke [49,50,51,52], within ischemic areas at post-stroke day 7. It remains unclear how pericytes are increased after reperfusion; however, cell niches around pericytes such as surviving endothelial cells may contribute to the production of pericytes within ischemic areas. In support of this idea, previous studies showed that brain endothelial-cell-derived trophic factors, such as PDGFB and basic fibroblast growth factor, promote the induction of PDGFRβ^+^ pericytes within ischemic areas after stroke [19,20].

Similar to endothelial cells, brain pericytes regulate the transmigration of inflammatory cells, including neutrophils [53]. In addition, pericyte depletion directly induces inflammatory responses in endothelial cells and perivascular infiltration of macrophages [54]. These findings indicate that pericytes have an important role in modulating inflammatory cells under inflammatory conditions such as after ischemic stroke. The effects of brain pericytes on inflammatory cells are not fully elucidated; however, pericytes secrete various factors (e.g., cytokine and chemokine) that can modulate the traits of macrophage/microglia into anti-inflammatory types [48]. Thus, the presence of pericytes may promote phenotypic change of macrophage/microglia into the anti-inflammatory M2 type. In support of this hypothesis, compared with permanent ischemia, we found that early reperfusion following ischemia increased the number of anti-inflammatory CD206^+^ M2 macrophage/microglia within ischemic areas proportionally to pericyte number at post-stroke day 7 [25]. Similar to pericytes [49,50,51,52], M2 macrophage/microglia are also associated with tissue repair [55,56,57]. Therefore, an increase of M2 macrophage/microglia by early reperfusion may promote brain repair following ischemic stroke.

Admittedly, the precise origin of macrophage/microglia under ischemic stroke is unclear. They may be derived from tissue-resident brain macrophage/microglia [58] and/or circulating cells that migrated into ischemic areas [59]. In addition, recent studies including ours demonstrated that macrophage/microglia at the site of ischemic areas are, in part, brain pericyte derivatives [60,61,62]. In contrast, macrophage/microglia have the potential to produce a subset of pericytes [63,64]. Thus, although the relationships between pericytes and macrophage/microglia under ischemic stroke are not fully understood, they may be cooperatively involved in brain repair.

Ischemic stroke frequently induces neurodegenerative changes associated with the development of cognitive impairments, such as Alzheimer’s disease (AD) [65,66,67]. The mechanism by which AD manifests after ischemic stroke is not known, but it has been reported that the expression of tau protein and amyloid protein precursor processing genes, principle factors of AD, were increased in brain tissues of an animal model of ischemic stroke [68,69]. In addition, patients with AD have a decreased pericyte count [70], indicating that pericytes play an important role in the pathogenesis of AD. In fact, mice deficient in pericytes had amyloid angiopathy and cerebral β-amyloidosis because of decreased clearance of soluble amyloid β, causing development of tau pathology and early neuronal loss, and resulting in cognitive dysfunction [71]. Further, pericyte deficiency causes decreased blood flow [47,72,73] and amyloid β oligomers constrict capillaries in AD through signaling to pericytes, thereby leading to CBF reduction [74]. In contrast, pericyte implantation enhanced CBF and reduced amyloid β deposition in a mouse model of AD [75]. Therefore, compared with permanent ischemia, early reperfusion after ischemic stroke may promote the maintenance of brain pericytes, in part, by increasing CBF, and thus, contributing to a decrease of neurodegenerative changes including the incidence of AD. Nevertheless, further experiments are needed to explore this hypothesis.

### 3.4. Reperfusion and Neural Regeneration

In mice, pericyte ablation induces a rapid reduction of neurons, in part, via loss of pericyte-derived neurotrophic growth factor [47], a finding which indicates that pericytes play an essential role in not only neural degeneration, but also neural repair following injury. The precise origins and traits of vascular pericytes in ischemic areas remains unknown; however, it has been reported that pericytes have the plasticity to generate various types of cells, including neural lineages, adipocytes, osteoblasts, chondrocytes, and myofibroblasts [76,77,78,79]. Thus, alteration of the environment within and/or around ischemic areas (e.g., oxygen concentration, tension against blood vessels, induction of chemokines, and infiltration of inflammatory cells) may affect the phenotype of brain pericytes subjected to ischemia/hypoxia. In fact, previous studies have demonstrated that brain pericytes within ischemic areas transdifferentiated into fibroblastic-like cells following stroke [49]. It is controversial whether brains harbor fibroblasts/fibroblast-like cells [80]. However, in other organs (e.g., skin, lung, and liver), it is well known that upon injury, mesenchymal cells will generate fibroblast-like cells (e.g., myofibroblasts), which promote healing because of their lasting contractile activity [81,82]. Thus, early reperfusion might increase production of not only pericytes, but also myofibroblasts, effectively decreasing the size of ischemic areas via mechanical forces. Whether a pericyte-mediated fibrotic response following brain injury contributes to neural repair and functional recovery is still under debate [83,84]. However, increasing evidence shows that fibroblastic-like cells derived from brain pericytes produce trophic factors and promote neural repair by promoting astrogliosis and oligodendrogenesis following ischemic stroke [16,85], suggesting that they provide a beneficial nursing effect for post-ischemic brains.

There is also increasing evidence showing that various types of stem cell populations such as neural stem/progenitor cells (NSPCs) reside in the central nervous system [86,87]. The precise origins and traits of stem cells that contribute to neural repair remain unclear; however, a growing body of evidence indicates that brain pericytes can behave as NSPC-like cells under pathological conditions such as those observed after ischemic stroke [21,22,62]. In addition, we demonstrated that brain pericytes, which were likely neural crest derivatives, acquired multipotency following ischemic stroke, presumably through mesenchymal-epithelial transition [21,60]. These ischemia/injury-induced multipotent stem cells (iSCs) expressed not only pericytic markers (e.g., PDGFRβ, neural/glial antigen 2 (NG2), and alpha-smooth muscle actin (αSMA)), but also various stem cell markers (e.g., nestin, c-myc, Klf4, and Sox2).

Further, accumulating evidence shows that pericytes have similar traits to those of mesenchymal stem cells (MSCs) [51,88,89]. Akin to iSCs, MSCs also have multipotent stem cell activity. MSCs reside in multiple organs, including bone marrow, umbilical cord, amnion, and brain [90,91,92,93]. Although it remains unclear whether brain pericytes are identical to brain MSCs [94], it was reported that the traits of MSCs differ among organs [95]. In support of this finding, we recently showed that compared with bone marrow-derived MSCs, iSCs, which likely originated from brain pericytes, predominately had neural rather than mesenchymal lineages, although they possessed several common pericytic (PDGFRβ, NG2, and αSMA) and mesenchymal markers (CD29, CD44, CD73, CD105, CD106, and CD166) [96,97,98,99]. We also demonstrated that iSCs were present in post-stroke human brain [99] and that iSCs obtained from mouse [98] and human brains [96,97] differentiate into electrophysiologically functional neurons. These results indicate that factors regulating the fate of endogenous iSCs (e.g., factors promoting cell proliferation of iSCs or factors inhibiting cell death of iSCs) can facilitate neural repair and may be clinically useful in treating patients with stroke [100].

Although the factors modulating stem cell populations induced under ischemic stroke are not fully known, we previously demonstrated that endothelial cells promote proliferation and neuronal differentiation of nestin^+^ stem cell populations, which presumably contained iSCs and/or NSPCs, following ischemic stroke [23,24]. In addition, we found that, compared with permanent ischemia, reperfusion during stage II increased the number of iSCs and/or NSPCs within and around ischemic areas, and promoted the production of neurons, astrocytes, and oligodendrocytes [25]. Therefore, early reperfusion has the potential to promote healing processes, including neurogenesis and gliogenesis via various vascular cell-mediated mechanisms, such as endothelial-derived nursing effects [23,24], pericyte-derived nursing effects [16,85], and pericyte-derived transdifferentiation into neural lineages [21,22,62]). Furthermore, compared with permanent ischemia, immunohistochemical findings at the chronic phase (post-stroke day 56) indicate that reperfusion after lethal ischemia increased the number of not only vascular lineages (including endothelial cells and pericytes), but also neural lineages (including neurons and glia) [25]. These enhanced reparative findings, such as vasculogenesis and neurogenesis, correlated with better behavioral outcomes at post-stroke day 56. Importantly, these findings indicate that reperfusion can be a beneficial treatment, even after lethal ischemia with mature neural cell death, potentially because of its initial therapeutic ability based on vascular cell-mediated healing processes (Figure 1).

Regarding behavioral tasks, we found that, compared with permanent ischemia, reperfusion during stage II improved the degree of depression-like symptoms evaluated by the forced swim test at post-stroke day 56 [25]. During the chronic period, patients with stroke frequently accompany various symptoms such as depression (termed post-stroke depression). Although the precise mechanism underlying post-stroke depression is unclear [101], our finding indicates that reperfusion during stage II has the potential to reduce the complication of post-stroke depression in patients with stroke during the chronic period.

## 4. Future Perspectives on Reperfusion Therapies Following Ischemic Stroke

We reported that although an ischemic insult of short duration was sufficient to induce neural cell death, vascular cells could still survive within ischemic areas, even after establishment of permanent ischemia [25]. These findings indicate that while the duration of stage I ischemia appears short, that of stage II is likely longer than previously thought. These findings also indicate that although some patients with stroke cannot receive the benefits of reperfusion during stage I, in part because of the narrow therapeutic time window, many patients with stroke are potentially retained within stage II, and thus, can potentially receive the beneficial effects of reperfusion via the activation of the healing process through vascular cells. We applied a 90-min duration of ischemia in mice and observed that reperfusion provided beneficial effects without increasing critical complications, including instances of mortality, hemorrhagic transformation, and BBB permeability [25]. Furthermore, other groups demonstrated that such beneficial effects of reperfusion after lethal ischemia were also observed in mice after longer ischemic insults (e.g., ischemia for 240 min) [16]. However, as the duration of ischemia increases, the BBB may exhibit more extreme damage, thereby increasing the frequency of these complications. In fact, a previous study showed that although early reperfusion decreased BBB permeability, it increased the incidence of hemorrhagic transformation in patients with stroke [35]. Therefore, the effects of reperfusion during stage II varies, according to the time until reperfusion after stroke onset, and should be explored in future studies.

Using a highly reproducible stroke model based on CB17 mice, which have fewer cerebral arterial branches and collateral vessels than C57BL/6 mice [30], we and others demonstrated that ischemia with a short duration (60 min or more) is adequate to cause lethal ischemia with mature neural cell death [16,25]. However, compared with CB17 mice after ischemic stroke, the duration of ischemia necessary to induce lethal ischemia would be longer in the case of patients with stroke, as the human brain is likely supplied with more collateral vessels, a factor which can affect the time window from stroke onset to treatment [102]. In support of this hypothesis, patients with stroke who received reperfusion within a suitable time window (e.g., t-PA therapy within 4.5 h after stroke onset [103]; neuroendovascular treatment within 16 h or 24 h after stroke onset [104,105]) occasionally recover with no sequelae. In addition, BBB leakage was detectable in patients with stroke during the first 3 h after onset of symptoms [106], although it was identified only at 30 min after reperfusion following MCAO [107]. Moreover, accumulating evidence from animal studies shows that cellular responses, including degenerative and reparative events following ischemic stroke, differ according to one’s age [108,109,110,111,112]. Thus, it may be challenging to acquire evidence for the efficacy of reperfusion therapies after lethal ischemia in patients with stroke, as human samples can be influenced by factors such as age, sex, the presence of complicating diseases (e.g., hyperlipidemia and hyperglycemia), ischemic area, the size of the ischemic area, and time to reperfusion after stroke onset [34,113,114]. Therefore, compared with a mouse model of stroke, in the case of patients with stroke, it is more difficult to determine, with precision, the time window for induction of lethal ischemia with mature neural cell death. Further studies are warranted for better delineation and characterization of the beneficial time window for reperfusion therapy for patients with stroke.

## 5. Conclusions

In summary, we describe the application of reperfusion after stroke from the viewpoint of findings based on a recent mouse model of stroke. The aforementioned studies revealed that a vascular cell-mediated healing system functions within ischemic areas, even after lethal ischemia that is accompanied by mature neural cell death. Moreover, they demonstrated that early reperfusion during stage II assists in the preservation of endothelial cells, proliferation of pericytes and NSPCs, as well as promotion of vasculogenesis, neurogenesis, and neurological functional recovery without causing any serious side effects. The possible efficacy of therapies targeting stage II ischemia suggests that the therapeutic time window of reperfusion can be expanded to longer periods than the current one. Currently, technology for imaging human brains is making impressive strides. Therefore, although the detailed relationships between neuroimaging findings and histological patterns under ischemic stroke remain unclear, an understanding of these relationships or the development of methods to visualize the histopathological changes using neuroimaging at the single-cell level in human brain would be helpful for future consideration of the indication of reperfusion therapies for patients with stroke.
